# Increased renal cortical stiffness is associated with coronary artery disease severity in patients with acute coronary syndrome

**DOI:** 10.1097/MD.0000000000016464

**Published:** 2019-07-12

**Authors:** Abdullah Orhan Demirtaş, Atilla Bulut

**Affiliations:** Department of Cardiology, University of Health Sciences, Adana Health Practices and Research Center, Adana, Turkey.

**Keywords:** coronary artery disease, renal circulation, ultrasonography

## Abstract

Atherosclerosis is the primary etiological factor associated with acute coronary syndrome (ACS). Kidneys have a highly arterial vascular structure and are therefore commonly affected by atherosclerosis, including those affecting the coronary arteries. Renal shear wave elastography (SWE) is an ultrasonographic method, which provides reliable information regarding the condition of the renal parenchyma.

We investigated the relationship between SWE findings and the severity of coronary atherosclerosis.

We calculated the following: the renal cortical stiffness (rCS) evaluated via SWE, the renal resistive index, the renal pulsatility index, the acceleration time, and the mean Syntax score (SS). Patients with a mean SS <12 were categorized into a low-risk (LR) and those with a mean SS ≥12 were categorized into the high-risk (HR) group.

Our study included 132 patients—76 in the LR and 56 in the HR group. Creatinine, high-sensitivity C-reactive protein (hs-CRP), and rCS were significantly higher, but the glomerular filtration rate (GFR) was significantly lower in the HR group. The Hs-CRP (odds ratio [OR] 1.220), GFR (OR 0.967), and rCS (OR 1.316) were observed to be independent predictors for the HR group. The cutoff value of rCS using receiver-operating characteristic curve analysis was 4.43 for the prediction of HR patients and showed 60.7% sensitivity and 57.9% specificity (area under the curve 0.642).

SWE which shows renal parenchymal injury and atherosclerosis in renal vessels may give an idea about the severity of coronary atherosclerosis.

## 1 Introduction

Coronary artery disease (CAD) is becoming increasingly widespread globally. Acute coronary syndrome (ACS) can show poorer prognosis than that observed with other forms of CAD in the absence of appropriate treatment.^[1,2]^ Atherosclerosis is the primary etiological factor associated with ST-segment elevation myocardial infarction (STEMI)-ACS and non-ST segment elevation myocardial infarction (NSTEMI)-ACS.^[1]^ Atherosclerosis is a systemic inflammatory process that can affect nearly all arteries in the human body. Cholesterol crystal deposition and local inflammation between the arterial intima and media layers play a primary role in the development of atherosclerosis.^[3]^ Following the onset of atherosclerosis, arterial wall elasticity diminishes in the presence of accompanying risk factors like hypertension, diabetes mellitus, and smoking with the eventual development of tissue oxygen supply imbalance.^[4,5]^

**Figure 1:**
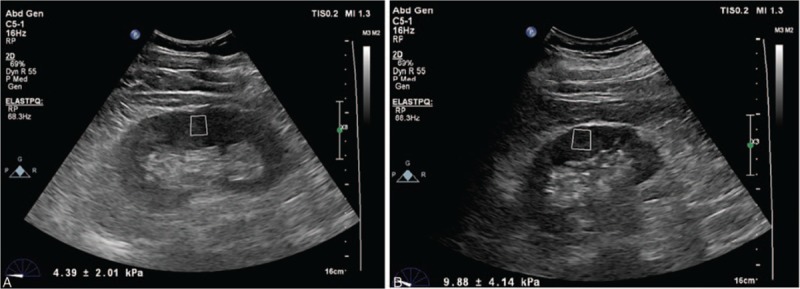
(A B) Images demonstrate a patient with standard shear wave elastography findings and increased shear wave elastography findings.

The brain and the kidneys have a highly arterial vascular structure and are therefore affected by atherosclerosis like the coronary arteries.^[6,7]^ Atherosclerosis causes important anatomical and functional changes in the kidneys.^[7,8]^ These functional changes can be monitored via the estimation of blood levels of electrolytes, blood urea nitrogen, creatinine, and urine osmolarity. Abnormalities in these laboratory parameters suggest the onset of irreversible damage to the kidneys. It has been shown that ultrasonographic (USG) methods can predict the development of such damage before such irreversible renal parenchymal impairment actually occurs.^[9]^ Renal shear wave elastography (SWE) is an ultrasonographic method, which provides reliable information regarding the condition of the renal parenchyma (Fig. 1A and B).^[10]^ It has been shown that renal cortical stiffness (rCS) evaluated using SWE is related to several types of renal parenchymal disease and fibrosis.^[11–13]^

The Synergy Between Percutaneous Coronary Intervention With Taxus and Coronary Artery Bypass Graft Surgery (SYNTAX) score (SS) is a comprehensive scoring system used to assess the severity of CAD.^[14]^ The SYNTAX score is an independent predictor of cardiac mortality in patients with ACS.^[15]^ Atherosclerosis can develop simultaneously in the coronary and the renal arteries. Renal atherosclerosis can cause renal parenchymal deterioration. The association between rCS and coronary atherosclerosis remains unclear in the literature.

We aimed to investigate the association between rCS and the severity of coronary atherosclerosis.

## 2 Methods

### 2.1 Patient population

We investigated consecutively hospitalized patients in the coronary intensive care unit with STEMI or NSTEMI between January 2017 and January 2018. The study protocol was approved by the Ethics Committee of the Cukurova University, Faculty of Medicine. Informed consent was obtained from each patient. This study protocol conforms to the Ethical Guidelines of the 1975 Declaration of Helsinki as reflected in a priori approval by the Institutional Human Research Committee. Patients with functional and anatomical renal disorders, disorders of hepatic function, previous percutaneous coronary intervention, or a history of coronary artery bypass surgery were excluded. Demographic data of patients were recorded.

### 2.2 Evaluation of laboratory findings

Blood samples were obtained in a routine manner, and the following were evaluated: blood glucose level, complete blood count, renal function tests, glomerular filtration rate (GFR), creatinine clearance (CrCl), lipid parameters, high-sensitivity troponin T (hs-TnT), creatine kinase-myocardial band (CK-MB), high-sensitivity C-reactive protein (hs-CRP), and uric acid level.

### 2.3 Echocardiographic assessment

The ejection fraction was recorded echocardiographically (Epiq 7 Philips Healthcare, DA Best, Netherlands).

### 2.4 Renal USG

Renal USG was performed in all patients 48 hours after hospitalization. A high-resolution Doppler ultrasound system (Philips EPIQ 7) equipped with a high-resolution convex probe (2–5 MHz) (Philips Health Care, Bothell, WA) was used. All patients had at least a 6-hour fast and a 20-minute rest before undergoing the USG. Gray scale abdominal USG was initially performed and quantitative Doppler parameters were subsequently recorded. Doppler USG measured the peak systolic velocity (PSV), end-diastolic velocity (EDV), and the acceleration time (AT) from both interlobular renal arteries at the intercostal window. The Doppler angle was maintained between 30 and 60 degree. After recording the PSV and the EDV, the spectral waveform was traced manually, and the renal resistive index (RRI) was calculated using the formula: PSV-EDV/PSV. The renal pulsatility index (RPI) was measured using the spectral waveform tracing and the PSV-EDV/mean flow rate formula. AT was defined as the time between the beginning of the systolic upstroke and the first systolic peak. All parameters were measured thrice in both kidneys. The mean RRI, RPI, and AT values were recorded.

SWE was performed using 2 to 5 MHz convex probes that utilized the ElastPQ software based on acoustic radiation force impulse imaging-based technology. All measurements were performed as described earlier.^[10]^ The probe was maneuvered in a steady manner with minimal compression. Patients were instructed to hold their breath in full inspiration for a few seconds to minimize motion of the kidney. The patient was placed in the left and right lateral decubitus positions, and measurements were obtained after the preliminary identification of a target region of interest (ROI) on a conventional ultrasonographic image. The ROI was placed perpendicular to an area of the renal cortex that did not contain any detectable vasculature or cysts. The main axis of the ROI was set parallel to the axis of the renal pyramids (perpendicular to the surface of the kidney). Minimal transducer pressure was applied during imaging to avoid mechanical compression of the kidney. The examination procedure was repeated for the contralateral kidney. We obtained 6 accurate measurements in each kidney in each patient, and a median value was calculated. If measurement reliability was low, the result was recorded as 0.00 kPa. The result was expressed in kPa.

### 2.5 Coronary angiographic evaluation

Patients were administered with 70 to 100 U/kg heparin bolus and 300 mg acetylsalicylic acid (ASA) initially at the time of diagnosis.^[1]^ After this, either 180-mg ticagrelor or 600-mg clopidogrel were given, or patients were redirected to coronary angiography (CAG) laboratory. Coronary angiography (CAG) was performed through femoral or radial access using the Judkins technique. Two cardiologists individually evaluated the CAG images. The left main coronary artery and other coronary artery occlusions were categorically recorded. The SS and clinical SYNTAX score (cSS) were calculated after including vessels with a diameter >1.5 mm and >50% stenosis determined using CAG images (http://www.Syntaxscore.com). Culprit lesion was predilated, and an appropriately sized stent was implanted. Thrombolysis in myocardial infarction (TIMI)-3 flow was achieved in all patients. Tirofiban (loading dose: 25 μg/kg IV infused within 5 minutes, infusion after loading: 0.15 μg/kg/min IV for up to 18 hours) or abciximab (0.25 mg/kg IV bolus over at least 1 minutes and 0.125 μg/kg/min IV continuous infusion for 12 hours) infusion was given to patients with high thrombotic burden. As in previous study ,^[14]^ the mean and median SS values were calculated for all patients. Patients with a mean SS <12 were categorized into a low-risk (LR) and patients with a mean SS ≥12 were categorized into a high-risk (HR) group.

### 2.6 Statistical analysis

Variables were classified into the categorical and continuous groups. Categorical variables were expressed as numbers and percentages. The *χ*^2^ test was used to analyze categorical variables. Continuous variables were expressed as means ± standard deviation. The Kolmogorov-Smirnov test was used to determine whether the continuous variables were normally distributed. Normally distributed variables were analyzed using the independent samples *t* test. Nonnormally distributed variables were analyzed using the Mann-Whitney *U* test. Independent predictors for HR patients were determined using binomial logistic regression analysis using *P* < .05 variables. Receiver-operating characteristic curve (ROC) analysis was used to calculate the cut-off, sensitivity, and specificity values for predictors of HR. The SPSS for Windows Program software version 20.0 (SPSS, Chicago, IL) was used for statistical analysis. A *P* value <.05 was considered statistically significant.

## 3 Results

**Figure 2:**
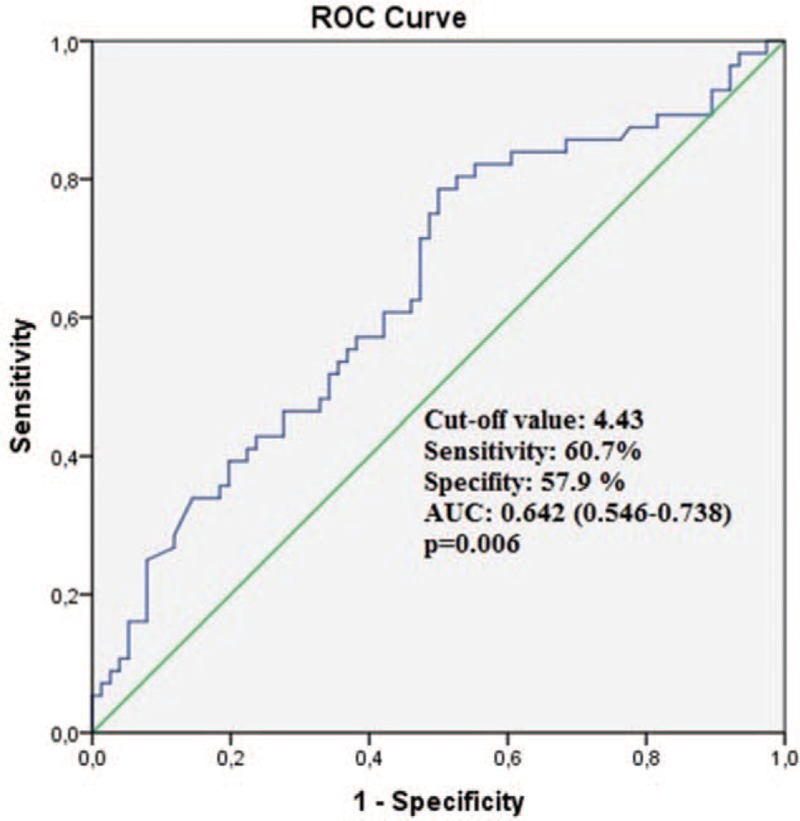
ROC analyses for renal cortical stiffness. AUC = area under the curve, ROC = receiver-operating characteristic curve.

Comparisons of LR and HR patients: Our study included 132 patients. The LR group included 76 patients (mean age 60.7 ± 12.9 years), and the HR group included 56 patients (mean age 63.4 ± 11.9 years). No statistically significant intergroup differences were observed in terms of demographic variables (Table 1). A comparison of laboratory values showed that creatinine (*P* = .022) and hs-CRP (*P* = .001) were significantly higher and GFR was significantly lower (*P* = .001) in the HR group, and other variables did not show statistically significant differences (Table 1). Echocardiographic and USG evaluation showed that the HR group demonstrated significantly higher rCS values (*P* = .006). A comparison of angiographic findings revealed that the HR group showed a significantly higher frequency of 2- and 3-vessel disease, SS, and cSS and that the LR group showed a significantly higher frequency of single-vessel disease (*P* <.001 for all measurements, Table 2). Variables that were observed to be statistically significant using univariate analysis were subjected to binominal logistic regression analysis (*P* <.05). We observed that hs-CRP (odds ratio [OR] 1.220, 95% confidence interval [CI] 1.029–1.445, *P* = .022), GFR (OR 0.967, 95% CI 0.943–0.992, *P* = .009), and rCS (OR 1.316, 95% CI 1.075–1.611, p=0.008) were independent predictors for the HR group (Table 3). The cutoff value of rCS using ROC analysis was 4.43 for the prediction of HR group (sensitivity 60.7%, specificity 57.9%). The area under the curve was 0.642 (*P* = .006) (Fig. 2).

**Table 1:**
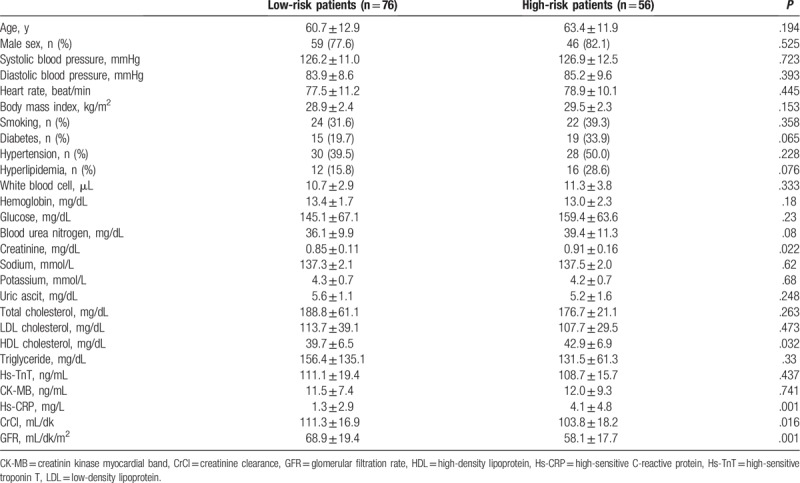
Comparison of patients demographic and laboratory findings.

**Table 2:**
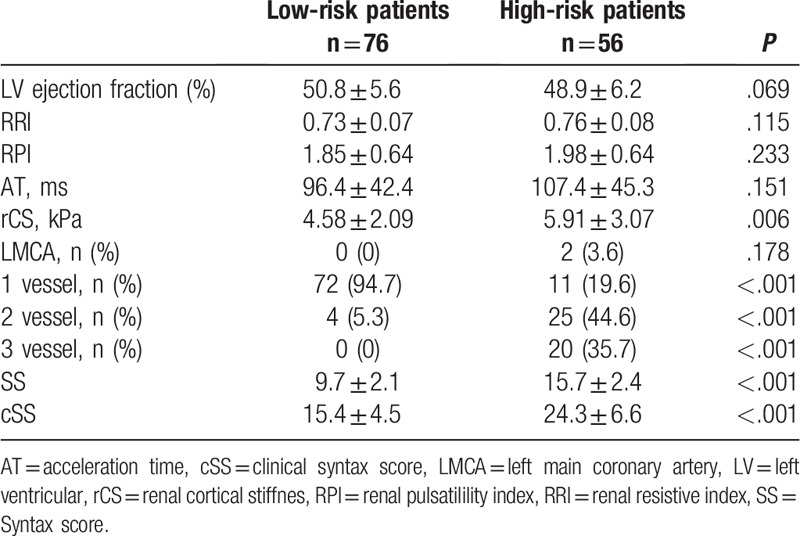
Comparison of patients echocardiographic, ultrasound and angiographic findings.

**Table 3:**
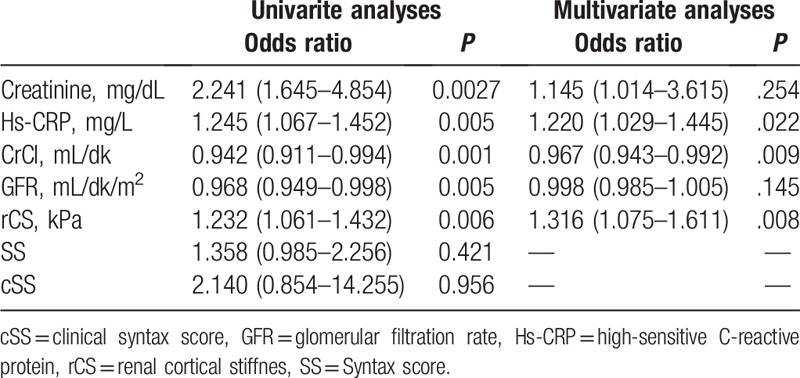
Independent predictors for high risk patients.

Comparison of STEMI and NSTEMI patients findings: NSTEMI patients were significantly older (P = .001), uric acid and hs-TnT were significantly lower (*P* values were .019 and .037, respectively), GFR values were significantly higher (*P* = .005, Table 4) compared to patients with STEMI patients.

**Table 4:**
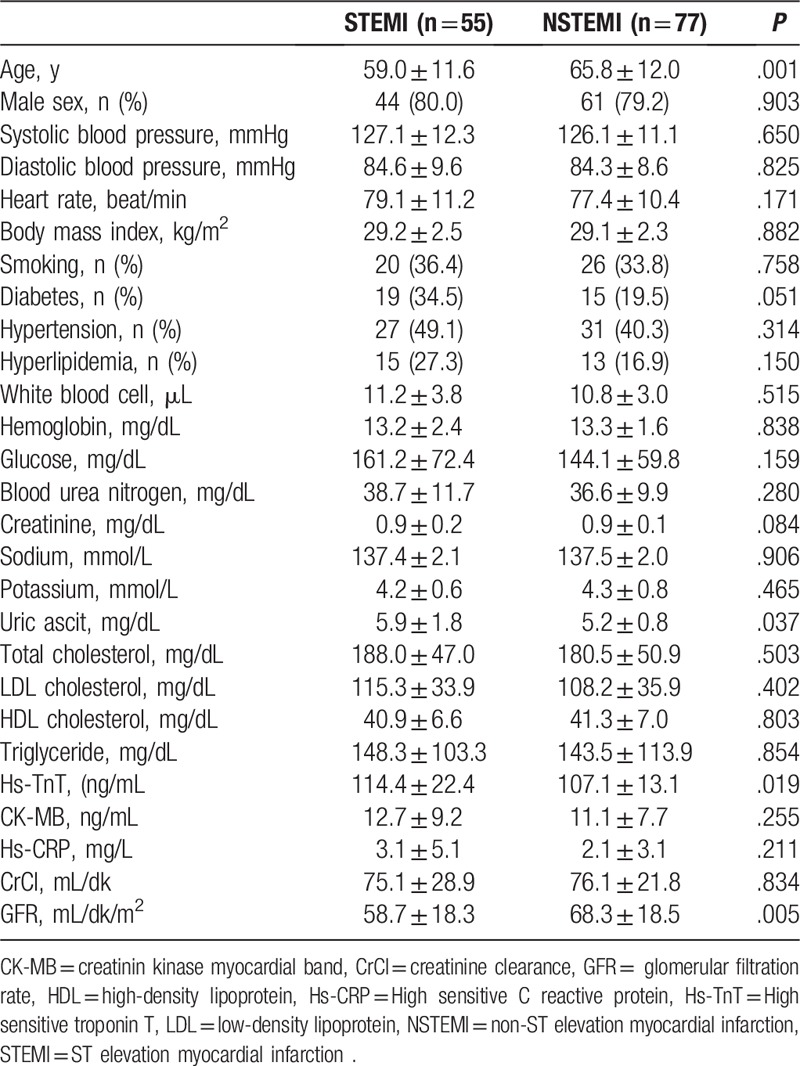
Comparison of STEMI and NSTEMI patients demographic and laboratory findings.

## 4 Discussion

To our knowledge, this is the first study to investigate the association between rCS and the severity of CAD. We observed that increased rCS was closely associated with CAD. Each unit increase in rCS increased the likelihood of severe CAD by 31.6%. Additionally, low GFR and high hs-CRP levels were observed to be associated with severe CAD.

The kidneys have a rich vascularized structure. They show anisotropic anatomy owing to a dual-compartment structure comprising the renal cortex and the medulla.^[9]^ Abnormalities of the vasculature including atherosclerosis, rupture, and/or compression markedly affect the elasticity of the kidneys. A study performed in animals has shown that sudden changes in blood flow essentially affected kidney elasticity.^[16]^ Lately, SWE is viewed as a noninvasive USG modality that enables detailed evaluation of renal tissue properties and rCS.^[17,18]^ No reference values have been conclusively documented for rCS. A meta-analysis involving small-scale studies reported that renal cortical elasticity was higher than that of the medulla.^[9]^ The authors reported a significant difference in rCS values between microalbuminuria and macroalbuminuria groups in patients with type 2 diabetes.^[19]^ A negative correlation was observed between rCS and estimated GFR in diabetic patients with kidney disease.^[10]^ These studies suggest a possible positive correlation between renal parenchymal damage and elasticity. Atherosclerosis can affect the entire renal vasculature. Renal parenchymal damage may occur secondary to atherosclerotic microvascular renal disease. Coronary atherosclerosis concurrent with atherosclerotic renovascular disease can cause increased. This information suggests a probable relationship between rCS and the severity of CAD. We observed a significantly increased rCS in the HR CAD group. This relationship was stronger than hs-CRP and GFR in our study. Also, we think that rCS increases in primary kidney diseases (such as glomerular, interstitial diseases or kidney stones). So, we excluded these patients from the study.

Recent studies show that the RRI obtained using Doppler USG was closely related to the severity of CAD.^[20,21]^ The RRI is a hemodynamic parameter, which can show rapid alterations. All patients undergoing the study should have at least a 6-hour fast and 20 minutres of rest before undergoing USG. The sensitivity of this evaluation is arguable to us because of its changeable nature by other parameters. We observed similar RRI values in our groups. Both groups in our study showed higher RRI values than those reported by other studies.^[20]^ Our study included patients with NSTEMI and STEMI. Therefore, the high RRI could be attributed to acute stress. Perhaps we might not have observed this difference in RRI if we had performed this evaluation after discharge.

### 4.1 Limitations of our study

Our study has relatively small and nonhomogeneous population (STEMI vs non-STEMI). USG evaluation of the kidneys is difficult owing to their retroperitoneal location. Moreover, evaluation is difficult in patients with obesity. All patients in our study showed a body mass index >25 kg/m^2^; thus, obtaining clear images of the kidneys was difficult. SWE performed using acoustic radiation force impulse imaging technology shows limited efficiency in kidneys that are located deeper than 5 cm. All our patients were diagnosed with CAD. This kind of study should ideally have been performed initially in patients with subclinical atherosclerosis. Further studies are warranted to support our hypothesis.

## 5 Conclusion

A close relationship is observed between the severity of CAD and rCS. Increased rCS values indicate a higher possibility of HR CAD.

## Author contributions

**Conceptualization:** Abdullah Orhan Demirtas.

**Data curation:** Abdullah Orhan Demirtas, Atilla Bulut.

**Formal analysis:** Abdullah Orhan Demirtas, Atilla Bulut.

**Funding acquisition:** Abdullah Orhan Demirtas.

**Investigation:** Abdullah Orhan Demirtas.

**Methodology:** Abdullah Orhan Demirtas, Atilla Bulut.

**Project administration:** Abdullah Orhan Demirtas.

**Resources:** Abdullah Orhan Demirtas.

**Software:** Abdullah Orhan Demirtas, Atilla Bulut.

**Supervision:** Abdullah Orhan Demirtas.

**Validation:** Abdullah Orhan Demirtas.

**Visualization:** Abdullah Orhan Demirtas.

**Writing – Original Draft:** Abdullah Orhan Demirtas, Atilla Bulut.

**Writing – Review & Editing:** Abdullah Orhan Demirtas, Atilla Bulut.

Abdullah Orhan Demirtas orcid: 0000-0003-4768-0536.
